# The ethics of crowdfunding in paediatric neurology

**DOI:** 10.1111/dmcn.15442

**Published:** 2022-10-21

**Authors:** Angus Livingstone, Laurent Servais, Dominic J. C. Wilkinson

**Affiliations:** ^1^ John Radcliffe Hospital Oxford UK; ^2^ MDUK Oxford Neuromuscular Centre, Department of Paediatrics University of Oxford Oxford UK; ^3^ Neuromuscular Reference Center University Hospital and University of Liège Liège Belgium; ^4^ Oxford Uehiro Centre for Practical Ethics, Faculty of Philosophy University of Oxford Oxford UK; ^5^ Murdoch Children's Research Institute Melbourne Australia

## Abstract

In the last decade, there has been a dramatic increase in the number of families resorting to internet‐based public appeals to fund access to novel, highly expensive, or experimental therapies for rare disorders. Medical crowdfunding may provide a means to fund treatments or interventions, but it raises individual and societal ethical questions. In this review, we consider the ethical challenges crowdfunding poses in paediatric neurology, drawing on the example of gene therapy for spinal muscular atrophy. We discuss physician responsibilities, and how neurologists should respond to crowdfunding that they encounter in clinical practice. We also briefly consider actions that can be taken by clinicians, charities, and crowdfunding websites to reduce harms. The best way to mitigate these harms may be to target the high costs and restrictive criteria that limit access to many novel treatments, and to optimize treatment utility, for instance by newborn screening.

**What this paper adds:**

- Crowdfunding is a social phenomenon arising from families' inability to access desired treatment.
- Treatments sought by crowdfunding range from those that are clearly beneficial (but unaffordable) to those that would be ineffective and potentially harmful.
- Crowdfunding carries a range of harms and risks to families and children and has wider social impact.

## What this paper adds

- Crowdfunding is a social phenomenon arising from families' inability to access desired treatment.
- Treatments sought by crowdfunding range from those that are clearly beneficial (but unaffordable) to those that would be ineffective and potentially harmful.
- Crowdfunding carries a range of harms and risks to families and children and has wider social impact.

**Abbreviation**
SMA, spinal muscular atrophy.

## WHY DO PEOPLE CROWDFUND?

Crowdfunding for medical treatment has grown significantly in the last decade.^1^ For example, GoFundMe, the leader in online medical crowdfunding, raised more than US$2 billion from 21.7 million donations to medical campaigns between 2016 and 2020.^2^

People crowdfund ultimately because of the financial burden of treatment. Medical care is frequently expensive, and crowdfunding may represent a means for individuals to meet costs. These fall into one of three distinct categories.

### Category 1: Standard of care treatment

Where treatments have demonstrated effectiveness and entered the treatment market, their costs may nevertheless prohibit access for many. In the USA, for example, an estimated 62% of bankruptcies are filed because of medical expenses,^3^ and hospitals actively counsel patients on setting up crowdfunding pages to help counter this.^4^ It may, therefore, be a symptom of an under‐resourced and inequitable health care system. However, in countries with public health care systems, crowdfunding campaigns still arise. In such countries, cost or cost‐effectiveness thresholds are used to identify when treatment may be funded. Where treatments are particularly expensive, inclusion criteria can be extremely stringent. Thus, people falling outside of these boundaries may turn to crowdfunding.^5^

One striking example is onasemnogene abeparvovec, or ‘Zolgensma’, a gene therapy that replaces the missing *SMN* gene in patients with spinal muscular atrophy (SMA). Often described as the most expensive drug in the world (at approximately US$2.1 million for a single dose),^6^ it is inaccessible to many. In patients identified presymptomatically, onasemnogene abeparvovec can be transformative and appears to allow many to remain symptom free.^7^ In patients treated after symptom onset, onasemnogene abeparvovec improves survival and reduces loss of motor function, but the benefit decreases significantly with symptom progression.^8^ This can result in significantly juxtaposed medical and societal costs.^9^ The high cost but potentially powerful benefit means that onasemnogene abeparvovec is prominent on crowdfunding websites. In May 2021, a search on GoFundMe identified 171 individual campaigns aiming to raise a total of US$256 million for onasemnogene abeparvovec.^10^

### Category 2: Treatment of uncertain benefit

In other cases, treatment may be unavailable because potential benefits are not clear. This is true of experimental drugs, which have little in the way of published scientific evidence to support their use. It may also apply to treatments in patient groups different from those previously evaluated.^5^, ^10^ The intended recipient may fall outside the inclusion criteria for funding from insurance or public health care systems, which have been set based on clinical trial data.

For example, for onasemnogene abeparvovec, a number of families resorted to crowdfunding before national funding bodies' approval. Even in health systems that now fund this treatment, parents may extrapolate benefits in different clinical situations. For onasemnogene abeparvovec, this could be extrapolating the benefits in presymptomatic or mildly symptomatic children to their own child who has already had a serious decline in motor function. Other parents seek onasemnogene abeparvovec as an addition to an alternative SMA therapy such as nusinersen, an intrathecally administered oligoantisense nucleotide. There is no study that demonstrates the superiority of onasemnogene abeparvovec over other gene therapies or the benefit from addition of a second therapy (the May 2021 search identified 13 GoFundMe pages seeking onasemnogene abeparvovec as an additional therapy for a child already receiving nusinersen).^10^

### Category 3: Non‐beneficial, non‐scientific treatment

In other cases, alternative or complementary treatments may be sought,^5^, ^11^, ^12^ despite evidence that they do not work (or having no scientific rationale for benefit).^13^ Usually, such treatments are excluded from public health care systems because of their inability to demonstrate clinical effects that differ from placebo.^13^ For example, a number of families have used crowdfunding for stem cell therapies for neurological conditions in children and adults.^14^ Treatments that are medically futile, or even actively harmful,^10^, ^15^, ^16^ may be sought by parents who are uninformed, misinformed, or simply desperate (particularly in the face of severe, life‐limiting illness in their child).

## WHAT ARE THE POTENTIAL ETHICAL CONCERNS WITH CROWDFUNDING?

### Costs of crowdfunding

Although crowdfunding is sought to pay for treatment, where campaigns succeed, side effects and/or additional required interventions may create further substantial financial burdens that then fall on public health care systems or add unforeseen burdens for parents. This includes circumstances where treatments are administered abroad; future costs may accrue to the original country if a child returns or later develops complications. Before the new and expensive medication era, the yearly cost of patients with SMA types 2 and 3 was estimated at between US$27 000 and US$82 000.^17^ This follow‐up cost is rarely factored into the crowdfunding appeal.

Paradoxically therefore, crowdfunding could increase rates of medical bankruptcies in countries with publicly funded health care where rates have been historically low, by encouraging families to seek costly treatment not covered by their health care system or insurance. They may then feel obliged or pressured to personally meet the financial shortfall where campaigns have not matched costs. In other cases, there may be significant resource implications for the health care system itself even where the financial costs have been met, for example, limited availability of intensive care beds for children requiring invasive respiratory support.

In these cases, questions arise as to the best uses of parents' limited money, time, and energy. Crowdfunding efforts may divert these resources away from other more practical or beneficial interventions, or from focussing on comfort and dignity where a child has a life‐limiting illness and other beneficial therapies do not exist.

### Privacy

Crowdfunding may compromise children's privacy. Crowdfunding pages that include more detailed information are more likely to receive donations.^18^ GoFundMe encourages detailed descriptions of the situation and wide sharing to social media.^4^, ^19^ One study of 25 pages crowdfunding for SMA from a scraped GoFundMe database found a median of 175 shares to Facebook per campaign.^20^ GoFundMe advises that photos should be personally identifiable and encourages seeking exposure through conventional media.^20^

A study across 100 crowdfunding campaigns on behalf of children found significant disclosure of personal information. Of these, 30% included the child's full name; 42% included their age; 40% discussed their daily struggles; and 52% had descriptions of their medical history, including past treatments, diagnoses, and symptoms. More than 90% had images of the child's face; in many of these, the child was receiving treatment (e.g. intubation).^20^

Occasionally, compelling cases can receive national and international media attention,^21^ amplifying this effect by several orders of magnitude. Once this information reaches the public domain, it may prove impossible to withdraw, and a surviving child may have to live with this information being available to friends, family, schools, potential employers, and others. This may be particularly significant in cases of socially stigmatized illnesses or for members of marginalized communities.^20^

While adults may judge benefits of crowdfunding to outweigh privacy risks,^22^ this is more problematic for crowdfunding involving children who are too young to be involved in the decision to sacrifice their privacy.

### Consent

Crowdfunding generates two separate consent‐related issues: consent to crowdfunding itself and consent to the treatment. Consent to crowdfunding raises distinctive questions regarding exploitation or deception. Families may be unaware of the aforementioned privacy or financial risks. Groups may capitalize on the availability of crowdfunding to take advantage of individuals who do not have the funds to pay for their treatment nor the medical literacy to independently decide on the best intervention. For example, there are cases of individuals crowdfunding for pay‐to‐participate trials for unproven interventions (such as stem cell treatments), lured by dubious or unproven claims regarding efficacy.^23^

Crowdfunding may introduce the donor into the decision‐making process. Recipients may feel pressure to make specific decisions based on what donors have donated for, even if circumstances change or new insights are gained.^18^ Where these cases receive significant media attention, this pressure potentially increases enormously. There is little evidence to estimate how often this occurs. In one prominent case, medical professionals that opposed a crowdfunded treatment received death threats.^18^

### Ethical issues for donors

Crowdfunding does not just affect campaigners, but also donors. Some pages may be obviously fraudulent,^4^ others better disguised, particularly if the page creator is medically literate.

In other cases, donors may be more subtly misled. Embellishing or exaggerating clinical or treatment details (purposefully or otherwise) may attract more donors.^3^ The May 2021 search revealed 20 out of 171 pages incorrectly referring to onasemnogene abeparvovec as a ‘cure’ for SMA.^10^ Another study investigated 408 crowdfunding pages and found a tendency to underestimate risks and exaggerate efficacy. These pages had been shared collectively 111 044 times on social media and had raised a total of US$1 450 011 from 13 050 donors, with a target of US$7 439 308.^16^ This issue may be greater still for alternative treatments.^11^ In some cases, donors may be unknowingly contributing to harmful interventions.^15^ This practice has the potential to spread misinformation, devalue the evidence base of medicine, and undermine the donor autonomy.^11^, ^12^, ^24^

Crowdfunding also benefits small numbers of identifiable individuals at the cost of much larger numbers of non‐identified people. It thus opposes principles of ‘effective altruism’. By their nature, the interventions sought in categories 2 and 3 represent an inefficient use of resources. Even in category 1, treatments can be highly costly relative to their benefit. (For example, the money for a single dose of onasemnogene abeparvovec could provide SMA newborn screening for 40 000 infants and identify four patients who could be treated presymptomatically).^9^ The benefit of donations is further reduced by margins deducted by crowdfunding websites. On the other hand, at least some of those donating to crowdfunding campaigns would not have otherwise donated their money. This may be particularly true for family or friends close to those crowdfunding, who may feel a personal moral duty to direct their money to the campaign. For those with no relationship to the patient who wish to do the most good with their donations, crowdfunding is unlikely to be the best way to do so.

Charities, meanwhile, may feel torn between supporting individual campaigns versus supporting scientific research or initiatives serving the wider community.^10^ They may feel pressure from their own donors to donate to particular campaigns. Pharmaceutical companies stand to gain free promotions for their products and can pressure public funders by demonstrating parents' treatment willingness.^10^

### Equality

Crowdfunding success is uneven, drawing resources from within existing social networks.^25^ For example, female gender and Black ethnicity are associated with strong disadvantages, with these groups raising 5.9% and 11.5% less in their campaigns respectively.^26^ Families in neighbourhoods with higher deprivation rates raise 26.1% less.^27^ Crowdfunding for routine care is less successful than individual interventions.^26^ Socially stigmatized illnesses may not be as compelling.^3^ Empathic illness narratives attract more donors, favouring those with higher literacy capabilities.^4^, ^27^ The technological divide plays a role; ability to access and use technology affects page maintenance and development.^4^, ^5^ Again, this may be compounded by media attention; individual compelling cases provide platforms for capitalizing on sensationalism and therefore attracting large numbers of donors to a single campaign. Herding, a common trend in behavioural economics, can further perpetuate this asymmetry.^28^

Another large‐scale analysis found that across 175 000 pages, crowdfunding was most effective in areas with high incomes and high levels of education.^29^ For onasemnogene abeparvovec in May 2021, only 33 out of 171 pages had reached more than 50% of their target.^10^

Crowdfunding advantages not those with the greatest need or potential benefit, but rather those with certain unique literacies, the most emotive photos and narratives, convincing stories, or best social networks. This in turn may widen inequities in access to medical care, and simultaneously divert attention away from the underlying causes, reducing impetus for reform.^18^, ^19^

Against these concerns, inequality exists regardless of crowdfunding. Even in the absence of crowdfunding, some patients and families can access treatments that others cannot. Removing the option of crowdfunding would make some worse off (for example because they would have no chance at all to access a treatment), without benefitting any others. Thus, while there may be systemic equity concerns related to crowdfunding, these are difficult to define at an individual level.

## SHOULD NEUROLOGISTS SUPPORT CROWDFUNDING?

Paediatric neurologists may become aware that their patients are crowdfunding or may be asked to provide support for campaigns. This may become increasingly common as novel and expensive therapies are developed for more conditions.

There is a potential ethical tension between clinicians' duties to individual patients and their wider ethical duties to promote equitable access to medical treatment. They may also experience conflict between supporting strongly desired initiatives that offer uncertain benefit and focussing on more realistic options where less stands to be gained.

In all cases, the involved clinician should (based on the principle of non‐maleficence) aim to minimize potential harms. They should provide clear, accurate information about the risks and benefits of both therapies and of crowdfunding. Where interventions will be clearly beneficial (category 1), there may be an ethical case for clinicians to support crowdfunding campaigns. Offering to review and ratify the claims made in the campaign narratives (for instance by confirming or editing the accuracy of claims for the crowdfunding page) may help engage families in conversation about the realistic risks and benefits of therapy and ensure that donors are provided with accurate information. By seeking consent, this can be done without compromising confidentiality.

In cases where it is clear that interventions will be futile, or indeed harmful (category 3), clinicians should not support crowdfunding and should instead focus on beneficial interventions, even where these constitute palliative measures rather than treatments. It is important to try to maintain a supportive relationship and to engage empathically with families' hopes and requests. Where outcomes are less clear (category 2), clinicians should encourage participation in research trials.

Where crowdfunding has already occurred, clinicians could face requests to administer treatments that are outside their usual practice. It is important to address parental expectations and understanding about the outcomes of therapy. Unrealistic expectations could result in refusal of other, more potentially beneficial interventions. Clinical ethics committee review may be helpful if the neurologist is unsure about whether to administer the requested therapy. Where the patient comes from overseas, it can be helpful to make plans in advance about ongoing care (potentially in their country of origin) to ensure both the best outcomes from treatment and management of any subsequent complications.

## SHOULD CROWDFUNDING BE REGULATED?

Although crowdfunding generates ethical concerns, regulating crowdfunding is challenging as it engages three basic freedoms: freedom of speech, freedom to seek non‐publicly funded health care, and the freedom to spend or donate money as desired.^10^ Furthermore, crowdfunding may provide genuine benefit to some families where treatment would be otherwise unavailable. Thus, as crowdfunding is likely to both continue and increase,^1^ we outline potential ways to reduce the ethical downsides of crowdfunding.

### Consent

Campaigners should seek explicit permission if crowdfunding on behalf of someone else's child.^20^ While parents and legal guardians of children have a right, within limits, to make decisions on their child's behalf, this right does not extend to close family members or friends. Seeking permission to do so does not necessarily reduce privacy loss, but at least promotes compliance with rights.

### Gatekeeping by crowdfunding sites

Crowdfunding sites have a responsibility not to promote harmful treatment or false advertising. In the past, certain crowdfunding sites have on occasion prohibited antivaccine campaigns because of their ‘promoting misinformation’, and campaigns for treatment at a clinic offering unproven cancer treatments.^12^, ^24^ This arguably does not constitute limitations on campaigners' free speech since crowdfunding sites are private companies.^24^ GoFundMe previously prohibited campaigns for products making health claims that have not been approved by applicable regulatory bodies but has since changed this policy to only prohibiting those that have been found by those bodies to cause harm.^24^ However, many treatments, particularly alternative treatments, have not been investigated, and thus will not be prohibited. We support proposals that companies like GoFundMe should revert to the previous policy.^24^ If health claims are ratified by regulatory authorities this would mitigate the risk of crowdfunding for unapproved uses. This will empower donors to make informed decisions, reduce the spread of misinformation, and limit consent‐based harms to campaigners. Alternatively, algorithms could down‐rank pages that seek funding for treatments of uncertain or absent benefit.

### Fraud

Likewise, more ought to be done by crowdfunding sites to remove fraudulent pages. It should be noted that GoFundMe claim that these pages make up less than 0.1% of pages and that swift action is taken to remove those that are fraudulent. However, the increase in crowdfunding's popularity coupled with the potential for those that are medically literate to create convincing pages could make this increasingly difficult. Requiring endorsement by a registered health professional may help to overcome this barrier.

### Equality

Crowdfunding companies and charities (for example those supporting children with neuromuscular disease) could increase accessibility, and actively promote and rank‐up campaigns from groups that have been previously disadvantaged. They could additionally provide packages of support and advice to such families.

## CONCLUSION

Ultimately, crowdfunding is a social phenomenon. It arises from health care systems, and is a symptom of families' inability to access desired treatment. It has wider impacts on the societies and communities to which children return. We have focused on the example of SMA, but this affects many conditions across paediatrics (and elsewhere). Although measures should be taken to reduce harms, the most effective action would be to address the underlying problem. Publicly funded health care systems and pharmaceutical companies need to work together to ensure that emerging treatments for rare conditions are affordable, and that families have access to trials for treatments not yet fully evaluated. More clarity on the costs of treatments and resulting treatment thresholds would potentially improve relationships with patients and could better rationalize crowdfunding decisions. Where clinicians become aware of families' crowdfunding, they should carefully assess the potential risks and benefits for the family and child.

## Data Availability

There are no data for this manuscript.
